# Effect of pressure on normal and superconducting state properties of iron based superconductor PrFeAsO_0.6_F_*y*_ (*y* = 0.12, 0.14)

**DOI:** 10.1038/s41598-017-11927-1

**Published:** 2017-09-15

**Authors:** S. Arumugam, C. Ganguli, R. Thiyagarajan, D. Bhoi, G. Kalai Selvan, K. Manikandan, A. Pariari, P. Mandal, Y. Uwatoko

**Affiliations:** 1 Centre for High Pressure Research, School of Physics, Bharathidasan University, Tiruchirappalli, 620 024 India; 2 ISSP, University of Tokyo, 5-1-5 Kashiwanoha, Kashiwa, Chiba, 277-8581 Japan; 3 Saha Institute of Nuclear Physics, HBNI, 1/AF Bidhannagar, Calcutta, 700 064 India

## Abstract

The effect of high pressure (up to 8 GPa) on normal and superconducting state properties of PrFeAsO_0.6_F_0.12_, an 1111-type iron based superconductor close to optimal doped region, has been investigated by measuring the temperature dependence of resistivity. Initially, the superconducting transition temperature (*T*_*c*_) is observed to increase slowly by about 1 K as pressure (*P*) increases from 0 to 1.3 GPa. With further increase in pressure above 1.3 GPa, *T*_*c*_ decreases at the rate of ~1.5 K/GPa. The normal-state resistivity decreases monotonically up to 8 GPa. We have also measured the pressure dependence of magnetization (*M*) on the same piece of PrFeAsO_0.6_F_0.12_ sample up to 1.1 GPa and observed *T*_*c*_ as well as the size of the Meissner signal to increase with pressure in this low-pressure region. In contrast, for an over-doped PrFeAsO_0.6_F_0.14_ sample, magnetization measurements up to 1.06 GPa show that both *T*_*c*_ and the Meissner signal decrease with pressure. The present study clearly reveals two distinct regions in the dome-shaped (*T*_*c*_-*P*) phase diagram of PrFeAsO_0.6_F_0.12_.

## Introduction

The discovery of superconductivity in iron-based compound LaFeAsO_1−*x*_F_*x*_ with high transition temperature, *T*_*c*_ ~ 26 K, has created a renewed interest in the field of superconductivity^1^. By replacing La with rare-earth elements (*Ln*) of smaller ionic size such as Ce, Pr, Nd, Sm and Gd, a new family of high-*T*_*c*_ superconductors emerges with transition temperature in the range of 26–55 K^2,3^. This behavior is very different from that observed in cuprate superconductors where *T*_*c*_ is found to be insensitive to ionic radius of the rare-earth ion. In pnictide compounds, the iron-pnictide (Fe-Pn) layer consisting of edge-sharing FePn_4_ tetrahedron is responsible for the occurrence of superconductivity and a strong correlation between the crystal structure and superconductivity is observed^4–6^. In *Ln*FeAsO (1111-type) superconductors, *T*_*c*_ reaches maximum when FeAs_4_ forms a regular tetrahedron, i.e., the As-Fe-As bond angle is close to 109.5°^4,6^. It has been suggested that the pnictogen height *h*_*Pn*_ measured from the Fe plane plays a crucial role for the *T*_*c*_ enhancement^6^. The pnictogen height is observed to increase and the FeAs_4_ tetrahedron tends towards regular shape as *Ln* changes from La to Gd and it acts as a switch between high-*T*_*c*_ state with nodeless paring and low-*T*_*c*_ state with nodal paring^6^. The effect of chemical pressure as well as external pressure on lattice structure and hence on superconductivity has been studied extensively to disentangle the relative contribution of various factors such as bond lengths, tetrahedral angle, and pnictogen height that affect superconductivity and provide valuable information to elucidate the mechanism of superconductivity in pnictides^4–12^.

Unlike chemical substitution, pressure is a continuously tunable thermodynamic parameter which can be used to understand the phase transition as well as charge conduction mechanism without introducing disorder in the system. There are several reports on the effect of pressure on superconductivity in pnictides^5,11–27^. However, the effect of pressure on *T*_*c*_ in *Ln*FeAsO_1−*x*_F_*x*_, in particular, in the low-pressure region, has not been unambiguously settled, possibly due to the sample quality and non-hydrostatic nature of the applied pressure. Also, the dependence of *T*_*c*_ on pressure is quite sensitive to doping level. In the low-pressure region, *T*_*c*_ shows an enhancement or suppression for 1111-type compound depending on the doping level and *Ln* ions^11–23^. In pressure dependent *ρ*(*T*) measurements, usually the superconducting transition is broadened and *T*_*c*_ has been mainly determined from the onset of resistive drop^11–16,18–23^. In contrast, measurements of magnetic susceptibility on *Ln*FeAsO_1−*x*_F_*x*_ compounds as a function of applied pressure has revealed that *T*_*c*_ is independent up to a threshold value of pressure and then decreases with further increase in pressure^24^.

Among the 1111-type compounds, though PrFeAsO_1−*x*_F_*x*_ system exhibits superconductivity at elevated temperature, the effect of pressure on superconducting and normal-state properties has not been studied systematically. In PrFeAsO_1−*x*_F_*x*_ system, the onset of superconductivity occurs at around 50 K for *x* = 0.12–0.15 composition^28–31^. Miyoshi *et al*.^24^ measured magnetic susceptibility on *Ln*FeAsO_1−*x*_F_*x*_ compounds and claimed that *T*_*c*_ is insensitive up to a threshold value of pressure and then decreases with further increase in pressure. For PrFeAsO_1−*x*_F_*x*_, it seems that *T*_*c*_ does not change from its ambient pressure value 43 K up to about 1 GPa and then decreases with the increase in pressure. However, the transition is not sharp in their sample possibly due to small superconducting volume fraction. Also, the interval of pressure increase used in their measurements is quite large to detect any small and systematic change in *T*_*c*_ with pressure in the low-pressure region.

In the present work, we have comprehensively studied the effect of pressure on normal and superconducting state properties of PrFeAsO_0.6_F_0.12_ sample, by measuring the temperature dependence of both resistivity and magnetization and on superconducting state properties of PrFeAsO_0.6_F_0.14_ sample from magnetization measurement. To reveal the behavior of *T*_*c*_ in the low-pressure region, we have measured resistivity and magnetization with a small step of pressure increase. Due to the sharp transition at ambient as well as applied pressure, the variation of *T*_*c*_ with pressure has been determined unambiguously. Irrespective of different criteria used for the determination of *T*_*c*_, it is observed that both *T*_*c*_ and Meissner signal increase with increase in pressure up to 1.1 GPa and *T*_*c*_ decreases above 1.3 GPa for PrFeAsO_0.6_F_0.12_. On the other hand, both superconducting transition temperature and the Meissner signal are observed to decrease with increase in pressure for the overdoped sample, PrFeAsO_0.6_F_0.14_.

## Results

**Figure 1:**
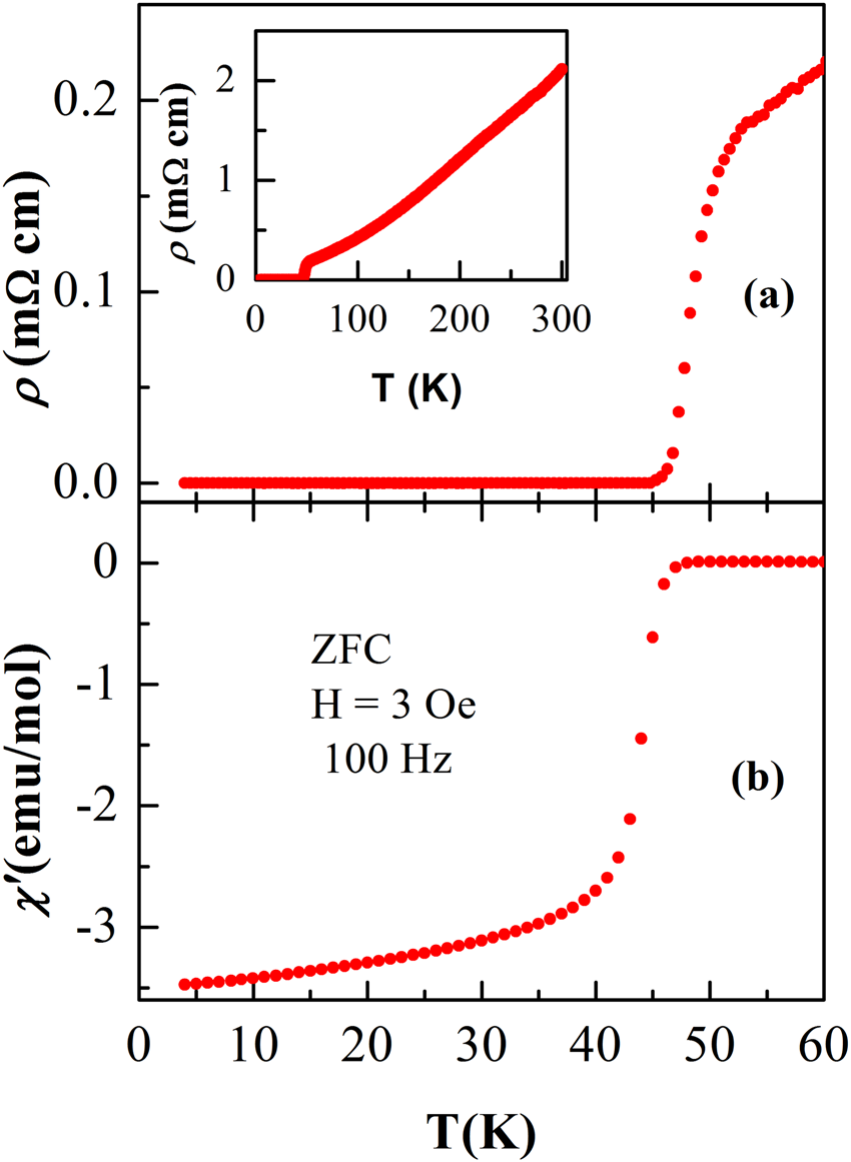
Temperature dependence of resistivity, *ρ*, (**a**) and the real part of the zero-field-cooled ac susceptibility, *χ*′, at *H* = 3 Oe (**b**) for PrFeAsO_0.60_F_0.12_ sample near the superconducting transition at ambient pressure. Inset of (**a**) shows the *ρ*(*T*) curve of the sample from 2 to 300 K at zero applied pressure.

Figure 1 shows the temperature dependence of resistivity and the real part of the zero-field-cooled (ZFC) ac susceptibility *χ*′ for PrFeAsO_0.60_F_0.12_ sample in the vicinity of the superconducting transition at ambient pressure. As shown in the inset of Fig. 1(a), *ρ* decreases from 2 to 0.16 m Ω cm as temperature decreases from 300 to 50 K and then drops sharply to zero due to the occurrence of superconductivity. It is also clear from Fig. 1(b), that *χ*′ starts to deviate from the normal behavior around 48 K due to the appearance of a diamagnetic signal, which is slightly above the zero-resistivity temperature. We observe that the superconducting volume fraction of this sample is quite large^31,32^. The sharp superconducting transition along with the large residual resistivity ratio (~13) and superconducting volume fraction indicate the good quality of the sample used in the present study. The details of sample characterisation are given in the Supplementary Information section (Figures S1 and S2).

**Figure 2:**
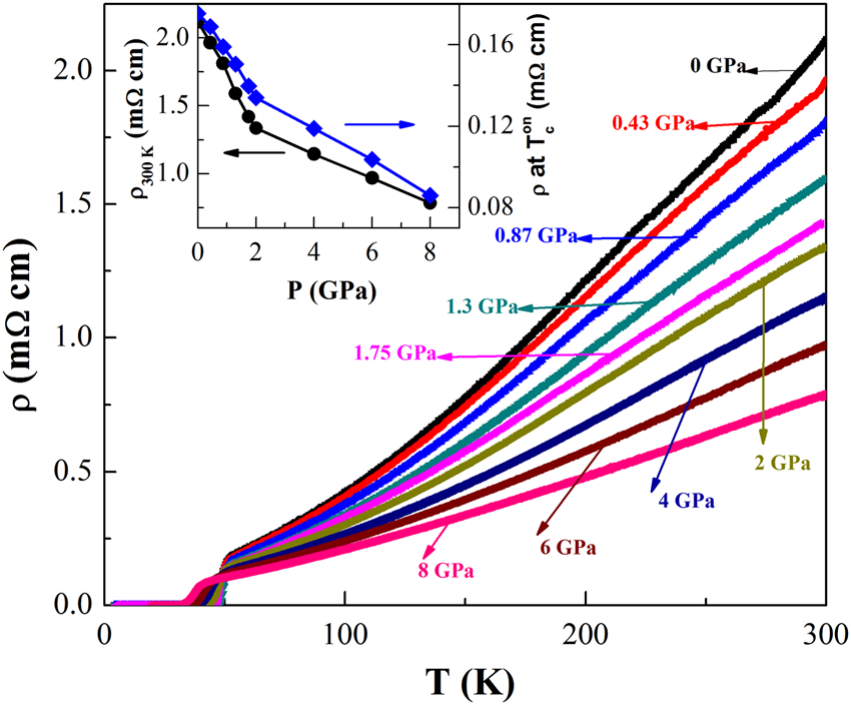
Temperature dependence of resistivity for the PrFeAsO_0.60_F_0.12_ sample under different applied pressures up to 8 GPa. The pressure is indicated by arrow. Inset shows the pressure dependence of the room-temperature resistivity (circle), *ρ*_*RT*_, and the value of the normal state resistivity (square) slightly above *T*_*c*_.

Figure 2 displays the temperature dependence of resistivity for the PrFeAsO_0.6_F_0.12_ sample under application of external pressure ranging from 0 to 8 GPa. The observation of sharp superconducting transition and zero resistivity at each applied pressure ensure the hydrostatic nature of the pressure in the series of measurements. Under compression, the overall value of resistivity decreases. With increase in pressure from 0 to 8 GPa, the room-temperature resistivity, *ρ*_*RT*_, decreases by a factor of 3; *ρ*_*RT*_ decreases almost linearly with a slope 0.4 m Ω cm GPa^−1^ up to 2 GPa while it decreases at a much slower rate 0.1 m Ω cm GPa^−1^ above 2 GPa. The normal-state resistivity slightly above *T*_*c*_ also follows a similar trend. It is clear from figure that the temperature dependence of *ρ* for PrFeAsO_0.6_F_0.12_ is very sensitive to the temperature region. *ρ*(*T*) is approximately linear in the high temperature region. With decrease in temperature, however, an upward curvature appears and this feature becomes quite prominent below 150 K. With application of pressure, the nature of temperature dependence of *ρ* gradually changes. The upward curvature in *ρ*(*T*) weakens progressively with increasing pressure.

**Figure 3:**
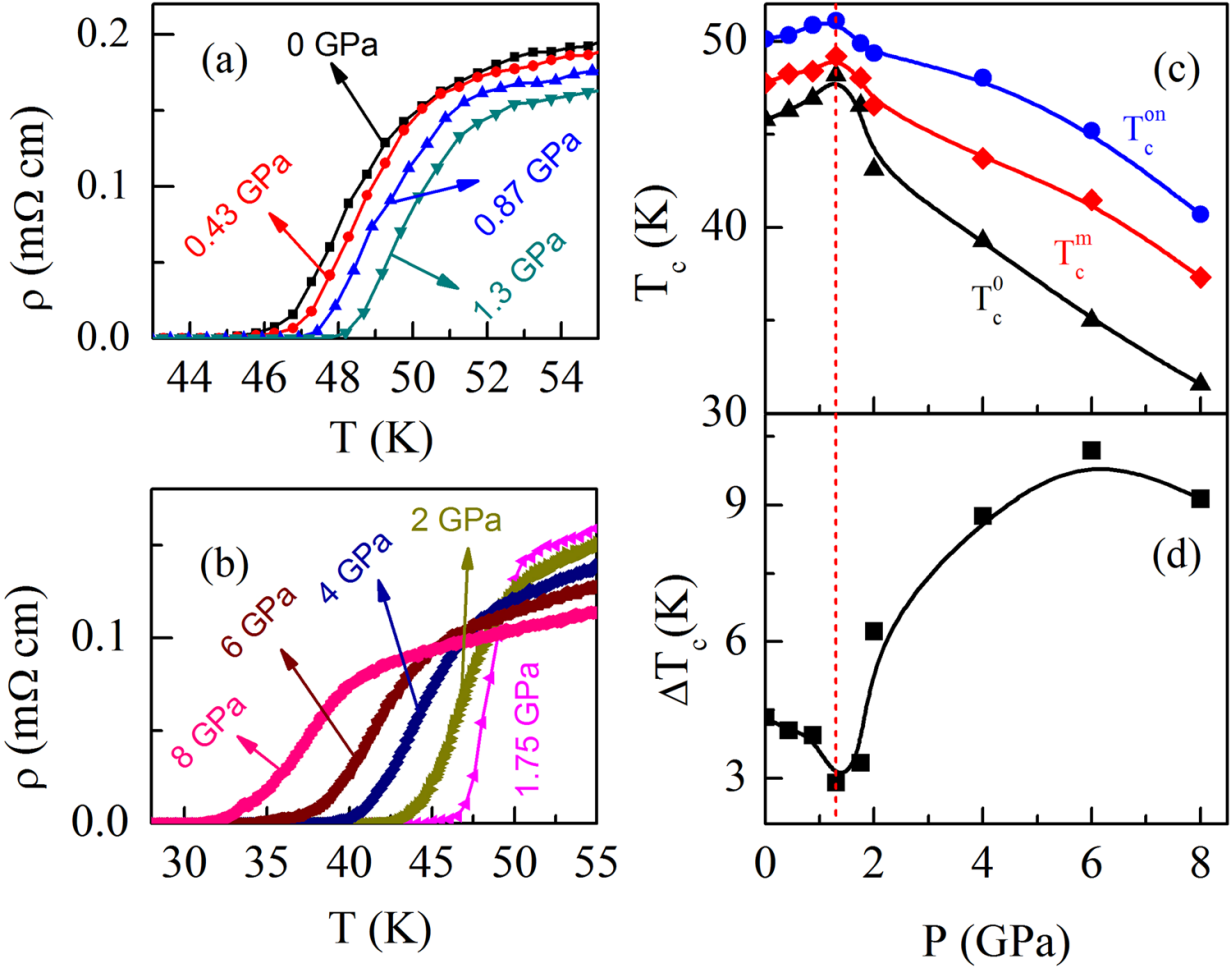
Pressure dependence of *ρ* near the superconducting transition for the PrFeAsO_0.60_F_0.12_ sample for: (**a**) pressure up to 1.3 GPa to show the increase of *T*_*c*_ and (**b**) above 1.3 GPa to show the decrease of *T*_*c*_. Pressure dependence of (**c**) the different superconducting transition temperatures (${T}_{c}^{on}$, ${T}_{c}^{m}$ and ${T}_{c}^{zero}$) estimated using the criteria as described in the text and (**d**) the width of the superconducting transition Δ*T*_*c*_ = ${T}_{c}^{on}$ − ${T}_{c}^{zero}$.

In Fig. 3(a) and (b), we have shown the evolution of superconducting transition with pressure. Up to 1.3 GPa, the superconducting transition shows a shift towards higher temperature [Fig. 3(a)]. However, for *P* > 1.3 GPa, the superconducting transition shifts to lower temperature as shown in Fig. 3(b). It is clear from the figure that the zero-resistivity state is achieved above 30 K even for the maximum applied pressure 8 GPa. To determine the effect of pressure on superconductivity unambiguously, we have used several definitions for the superconducting transition. Figure 3(c) shows the pressure dependence of *T*_*c*_ defined using different criteria, ${T}_{c}^{on}$, ${T}_{c}^{m}$ and ${T}_{c}^{zero}$. The superconducting onset transition temperature ${T}_{c}^{on}$ is determined from the intersection of the two extrapolated lines; one is drawn through the resistivity curve in the normal state just above the occurrence of superconductivity, and the other is drawn through the steepest part of the resistivity curve in the superconducting state. The midpoint ${T}_{c}^{m}$ is determined from the peak position of the d *ρ*/d *T* versus *T* curve, i.e., the temperature at which *ρ* exhibits a sharp change. ${T}_{c}^{zero}$ is determined from the zero resistivity. A striking feature is that all the *T*_*c*_s increase in the low-pressure region (*P* ≤ 1.3 GPa) and they decrease monotonically above 1.3 GPa [Fig. 3(c)]. It is important to mention that due to almost parallel shift of *ρ*(*T*) curve in the vicinity of superconducting transition, ${T}_{c}^{on}$, ${T}_{c}^{m}$ and ${T}_{c}^{zero}$ all vary with *P* in a similar fashion below 1.3 GPa [Fig. 3(c)]. However, the pressure dependence of the width of the superconducting transition, Δ*T*_*c*_ = (${T}_{c}^{on}$ − ${T}_{c}^{zero}$), shows an opposite trend. Up to 1.3 GPa, the transition width decreases slowly, and thereafter increases [Fig. 3(d)]. The decrease in Δ*T*_*c*_ in the low-pressure region may be partly due to the grain boundary effect. With pressure, intergrain coupling enhances, as a result, ${T}_{c}^{zero}$ is expected to increase and hence (${T}_{c}^{m}$ − ${T}_{c}^{zero}$) will decrease. The increase in sharpness of transition with pressure is also intrinsic, due to the enhancement of intragrain superconducting volume fraction. From the analysis of d *ρ*/d *T* versus *T* curve, we observe that (${T}_{c}^{on}$ − ${T}_{c}^{m}$) also decreases with increase in pressure, indicating the decrease in Δ*T*_*c*_ is partly intrinsic in nature. We would like to mention that qualitative similar behavior has been reported for FeSe single crystal^33^. With application of pressure, the onset of the superconducting transition gradually increases while the transition width, which is defined as the difference in temperature corresponding to 90 and 10 % of the normal-state resistivity, decreases in the low-pressure region (*P* < 0.8 GPa). This behavior has been attributed to the enhancement of superconductivity under pressure.

**Figure 4:**
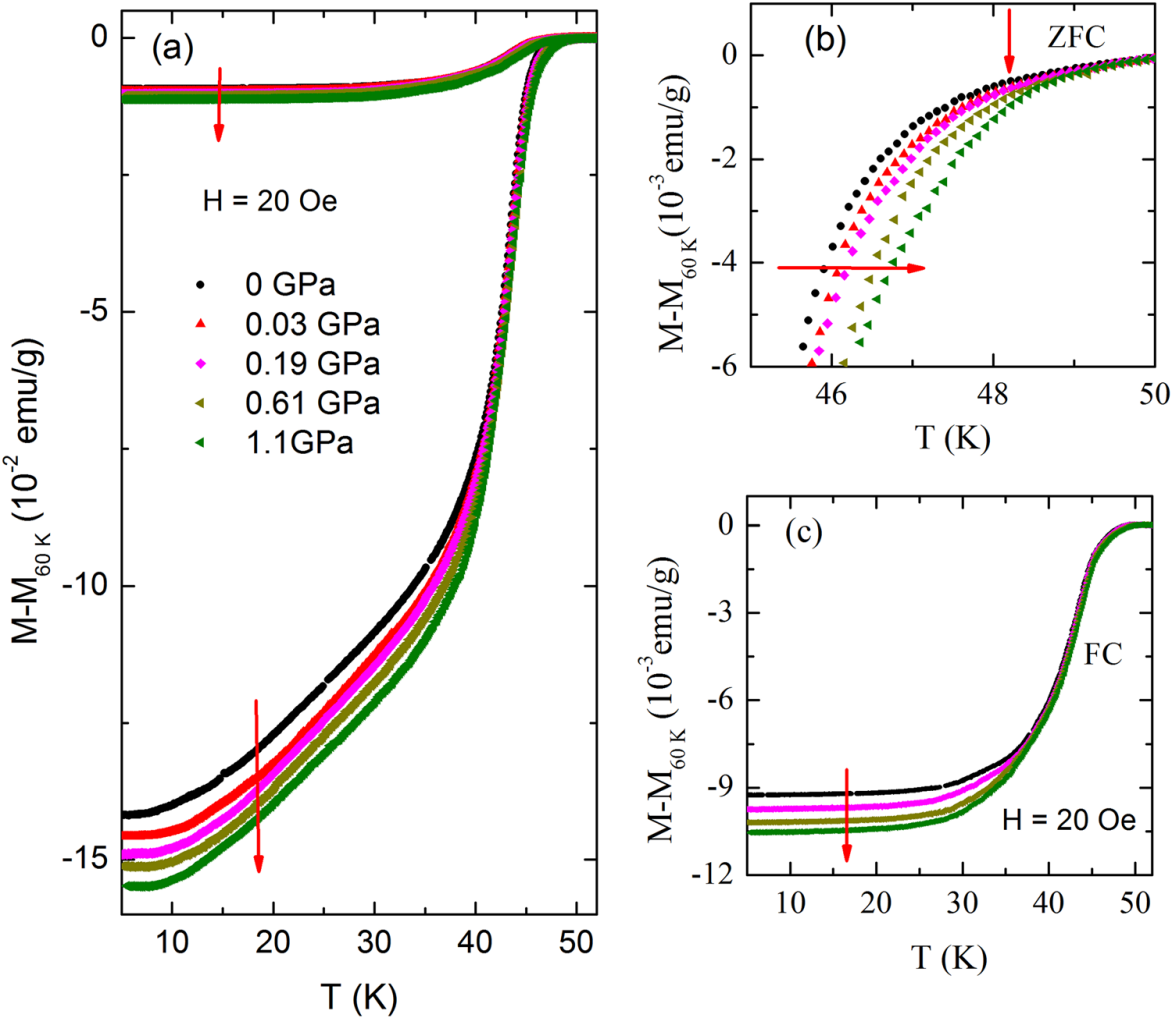
(**a**) Temperature dependence of field-cooled (FC) and zero-field-cooled (ZFC) magnetization for the PrFeAsO_0.60_F_0.12_ sample under different applied pressures up to 1.1 GPa. The downward arrow is to show the increase of Meissner signal with applied pressure. (**b**) The enlarged view of the ZFC magnetization curves in the vicinity of superconducting transition. The small vertical downward arrow indicates the ${T}_{c}^{on}$ at ambient pressure. The horizontal arrow represents the shifting of ${T}_{c}^{on}$ towards higher temperature with increasing pressure. (**c**) The downward arrow indicates the increase in Meissner signal with increasing pressure in FC magnetization measurement.

Measurement of pressure dependence of *T*_*c*_ solely from resistive data may not be sufficient to conclude that the deduced transition corresponds to bulk *T*_*c*_. To determine the pressure dependence of *T*_*c*_ in PrFeAsO_0.6_F_0.12_ unambiguously, we have also measured the Meissner effect on this sample for different applied pressures. The Meissner effect is an evidence of the bulk nature of the superconductivity. In Fig. 4(a), the temperature dependence of field-cooled (FC) and ZFC dc magnetization at different hydrostatic pressures up to 1.1 GPa is shown. With decreasing temperature, both FC and ZFC magnetization show a deviation from the normal-state behavior due to the onset of diamagnetism, signaling the occurrence of superconductivity. Further lowering the temperature below the superconducting onset temperature, magnetization drops sharply as in the case of resistivity. Thus superconducting transition under pressure remains very sharp both in resistivity and magnetization measurements. For clarity, the behavior of *M*(*T*) curve in the vicinity of *T*_*c*_ is expanded as shown in Fig. 4(b). It is clear from the plot that *T*_*c*_ increases monotonically with applied pressure up to 1.1 GPa. The shift of superconducting transition towards higher temperature indicates a positive pressure coefficient of *T*_*c*_ as in the case of resistivity. The superconducting transition temperature is defined as the temperature where the field-cooling magnetization starts to deviate from the normal behavior as shown in Fig. 4(b). When the applied pressure is increased from 0 to 1.1 GPa, *T*_*c*_ is found to increase by about 0.8 K which is close to that observed from resistivity measurements. Besides the pressure dependence of *T*_*c*_, it is important to investigate the effect of pressure on the Meissner signal. The temperature dependence of FC magnetization data in the low-temperature region has been expanded and shown in Fig. 4(c). One can see that the Meissner signal displays a systematic increase with pressure. Thus pressure dependence of magnetization study is fully consistent with the resistivity data. We would like to mention that the magnetic transition under pressure is much sharper in the present sample than the earlier reports.

Similar to other *Ln*FeAsO pnictides, in PrFeAsO, the orthorhombic to tetragonal structural transition and the antiferromagnetic phase transition of iron sublattice are progressively suppressed with the substitution of fluorine at oxygen site and superconductivity starts to appear when the fluorine concentration reaches a threshold value ~8%^30^. The long range antiferromagnetic ordering of Fe moments also disappears at this critical concentration. The superconducting transition temperature in PrFeAsO_1−*x*_F_*x*_ is observed to increase with *x* and reaches maximum of 47 K at around *x* = 0.15. With further increase of *x* up to 0.27, which is the maximum solubility of F in PrFeAsO, no significant change in *T*_*c*_ is observed, i.e., superconducting transition is insensitive in the overdoped region. However, a slightly enhanced *T*_*c*_ was reported in F-doped PrFeAsO prepared by different methods^28–31^. PrFeAsO_1−*x*_F_*x*_ synthesized using high-pressure technique and oxygen-deficient PrFeAsO_1−*x*_F_*y*_ exhibit maximum *T*_*c*_ ~ 50 K. In order to reveal the effect of pressure on doping, we have also prepared another oxygen deficient sample with slightly higher F content, PrFeAsO_0.60_F_0.14_. Although the amount of F content was not changed significantly, from the Hall resistivity measurement (Figure S3), we observe that the carrier density in PrFeAsO_0.60_F_0.14_ is about two times larger than that in PrFeAsO_0.60_F_0.12_. This suggests that PrFeAsO_0.60_F_0.14_ is in the heavily overdoped region of the phase diagram. We believe that such a large difference in carrier density between the two samples is mainly due to the significant deviation in oxygen vacancy from the nominal composition, i.e., the actual oxygen content in PrFeAsO_0.60_F_0.14_ can be lower than that in PrFeAsO_0.60_F_0.12_. The details on Hall measurements are presented in the Supplementary Information section.

**Figure 5:**
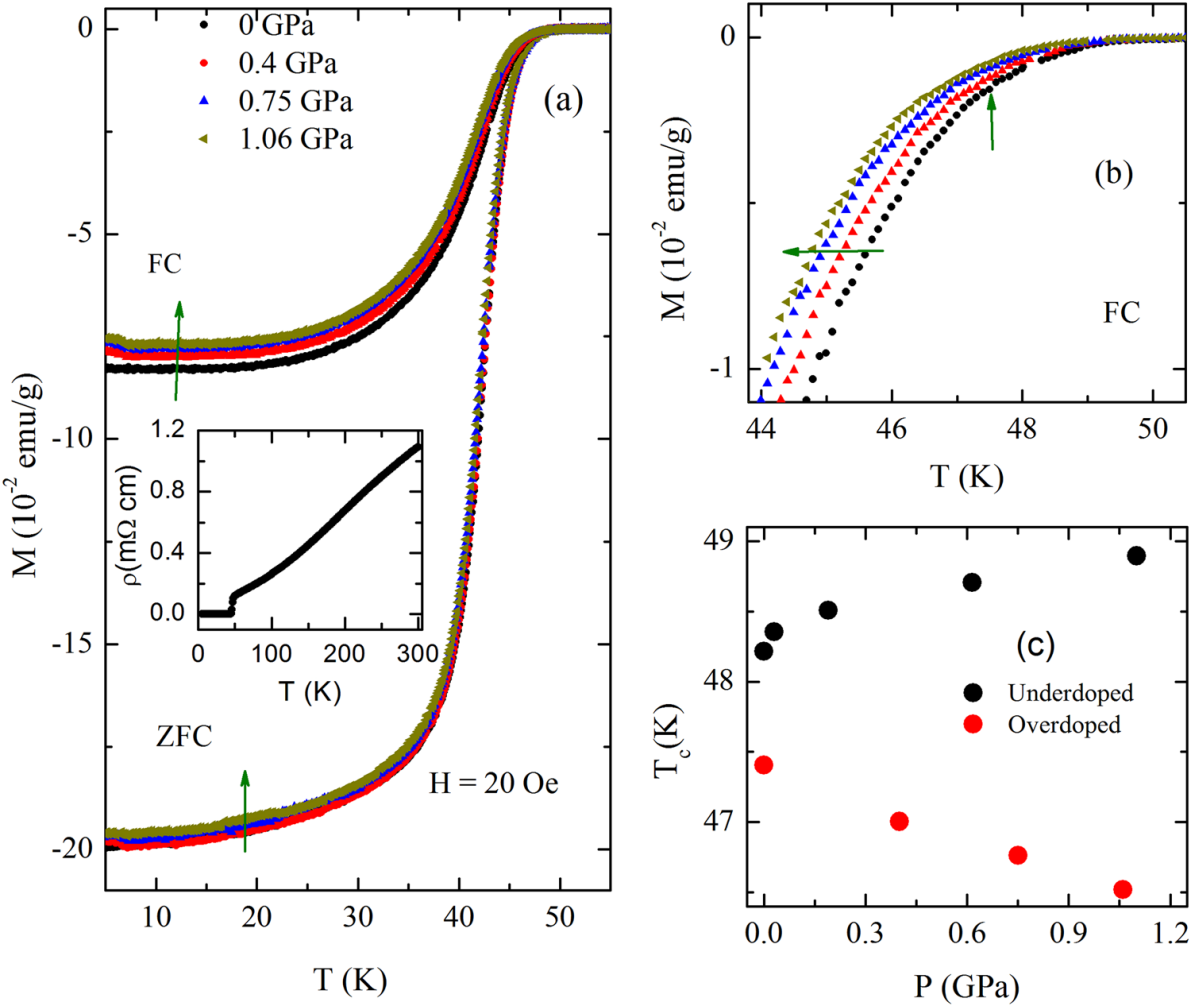
(**a**) Temperature dependence of field-cooled (FC) and zero-field-cooled (ZFC) magnetization for the PrFeAsO_0.60_F_0.14_ sample under different applied pressures up to 1.06 GPa. The upward arrow is to show the decrease of Meissner signal with applied pressure. Inset shows the temperature dependence of resistivity and superconducting transition at ambient pressure. (**b**) The enlarged view of the FC magnetization curves in the vicinity of superconducting transition. The horizontal arrow represents the shifting of ${T}_{c}^{on}$ towards lower temperature with increasing pressure. The small vertical upward arrow indicates the ${T}_{c}^{on}$ at ambient pressure. (**c**) The pressure dependence of *T*_*c*_ for both *y* = 0.12 and 0.14 samples.

For understanding the effect of pressure on superconducting-state properties in the overdoped region of PrFeAsO-based superconductor, we have also measured FC and ZFC magnetization under pressure for PrFeAsO_0.60_F_0.14_ sample. The temperature dependence of FC and ZFC magnetization at different hydrostatic pressures are shown in Fig. 5(a). The inset of Fig. 5(a) shows *ρ* vs *T* plot at ambient pressure. The value of *ρ* at room temperature is about half of that for the *y* = 0.12 sample, which is consistent with the deduced carrier density from the Hall resistivity. It is evident from both *M*(*T*) and *ρ*(*T*) curves that the superconducting transition for this sample is quite sharp as in the case of *y* = 0.12. However, the transition temperature is about 1 K lower. The transition region has been expanded as shown in Fig. 5(b). One can see that the pressure dependence of superconductivity is very different from that observed in *y* = 0.12 sample. For PrFeAsO_0.60_F_0.14_, superconducting transition progressively shifts towards lower temperature with increase in pressure up to 1.06 GPa. Unlike PrFeAsO_0.60_F_0.12_, Fig. 5(a) clearly shows a systematic decrease in Meissner signal with the application of pressure. The pressure dependence of *T*_*c*_ for both *y* = 0.12 and 0.14 is shown in Fig. 5(c). *T*_*c*_ increases almost linearly with pressure for *y* = 0.12 while it decreases with pressure for *y* = 0.14. The observed pressure evolution of superconductivity in PrFeAsO_0.6_F_*y*_ may be compared with SmFeAsO_1−*x*_F_*x*_ because the maximum *T*_*c*_ for these two systems is close 50 K^16,17^. In SmFeAsO_1−*x*_F_*x*_ too, with increase in pressure, *T*_*c*_ is found to increase for *x* ≤ 0.12 while it decreases above *x* = 0.12.

## Discussion

It is worthwhile to compare the present results with earlier reports on pressure dependence of *T*_*c*_ in 1111 compounds. In LaFeAsO_1−*x*_F_*x*_, the response of ${T}_{c}^{on}$ is positive up to a threshold value of pressure, regardless of the doping state of the sample, whether it is underdoped, optimally doped, or overdoped^13,15,20^. In these compounds, *T*_*c*_ versus *P* clearly reveals a dome shape curve, indicating the existence of two distinct pressure regions; the enhancing and suppressing of ${T}_{c}^{on}$ with increasing pressure. In optimally and overdoped LaFeAsO_1−*x*_F_*x*_ compounds, ${T}_{c}^{on}$(*P*) attains a maximum value 43 K, whereas for underdoped compound ${T}_{c}^{on}$(*P*) attains a maximum value 30 K^15^. The different features of *T*_*c*_(*P*) curve in the underdoped and overdoped compounds have been explained on the basis of the difference in their band structure. It has been suggested that for overdoped samples, pressure causes an increase in the density of states at the Fermi energy leading to a large enhancement of *T*_*c*_, whereas for underdoped samples pressure promotes the Fermi surface nesting induced antiferromagnetic fluctuations which in turn encumber the large enhancement of *T*_*c*_^20^. The effect of pressure on ${T}_{c}^{on}$ for Sm-based 1111 compounds are varied. Pressure can either suppress or enhance ${T}_{c}^{on}$ depending on the doping level. With increase in pressure, ${T}_{c}^{on}$ increases for the underdoped material whereas it decreases for the overdoped composition^16,17^. In contrast, Garbarino *et al*.^12^ found that in overdoped Sm-1111 compound, ${T}_{c}^{on}$ reveals a nonmonotonous pressure behavior with a maximum at 0.6 GPa, followed by a linear decrease at higher pressure. The increase in ${T}_{c}^{on}$ in the low-pressure region has been attributed to charge transfer to Fermi surface from the Sm 4 *f* orbital^12^. Compared to this, in Ce- and Nd-based compounds, a monotonous decrease in *T*_*c*_ with increasing pressure has been observed^14,21–23^. For Ce-1111 compound, *T*_*c*_ is suppressed to zero rapidly as compared to other 1111 compounds due to the increase in hybridization between Ce 4 *f* and Fe 3 *d* orbitals. In NdFeAsO_0.88_F_0.12_, with increasing pressure above 1.7 GPa, an upward curvature appears in *ρ*(*T*) curve in the low-temperature region and *ρ* follows a log *T* dependence, possibly due to the Kondo effect and *T*_*c*_ decreases linearly at the rate of 2.8 K/GPa^23^.

In most of the cases, the resistive transition is severely broadened, and sometimes the zero-resistance state can not be achieved even at a modest pressure ≲1 GPa. Due to the broadening of the resistive transition, it is extremely difficult to determine *T*_*c*_ precisely from the *ρ*(*T*) curve. In such cases, magnetization data, in which *T*_*c*_ determined from the onset of diamagnetism roughly matches with ${T}_{c}^{zero}$, may be used. Miyoshi *et al*.^24^ have investigated pressure dependence of magnetization for *Ln*FeAsO_1−*x*_F_*x*_ (*Ln* = La, Ce-Sm) samples. For *Ln* = La, *T*_*c*_ is found to be pressure independent up to about 3 GPa and, above 3 GPa, *T*_*c*_ shows a monotonic decrease. The width of the plateau decreases with the decrease in ionic radius of the rare-earth ion and shrinks almost to zero for *Ln* = Sm. We have already mentioned that diamagnetism does not appear sharply in their magnetic measurements on PrFeAsO_1−*x*_F_*x*_ at ambient as well as applied pressure. For this reason, the accurate determination of pressure dependence of *T*_*c*_ is not possible. The transition width is also found to be very sensitive to the nature of applied pressure. The nonhydrostaticity of pressure due to the solidification of the pressure transmitting medium at low temperature and high pressure may cause the broadening of the transition width. Several studies have shown that Daphne oil produces a good quality hydrostatic pressure with pressure up to about 3 GPa. For higher pressure, the cubic anvil press generates a homogenous pressure compared to the diamond anvil. Hence, our measurements on the present sample have eliminated the above problem, resulting in more trustworthy data. For further support on the pressure dependence of *T*_*c*_, we have measured magnetization on the same piece of PrFeAsO_0.6_F_0.12_ sample under hydrostatic pressure. Similar to resistivity, superconducting transition determined from magnetization measurements also shifts towards higher temperature with pressure at the rate of ~0.8 K/GPa which is very close to that observed in the case of resistivity. The increase in *T*_*c*_ and Meissner signal in PrFeAsO_0.6_F_0.12_ with pressure may be due to the enhancement of superfluid density. In Co-doped PrFe_1−*x*_Co_*x*_AsO (*x* = 0.075), both *T*_*c*_ and Meissner signal are found to increase with hydrostatic pressure up to 0.8 GPa which have been ascribed to the increase in superfluid density^34^. In underdoped cuprate superconductors, *T*_*c*_ is found to increase linearly with the ratio of superfluid density *n*_*s*_ to effective mass of carrier ${m}^{\bigstar }$, which is known as Uemura relation. However, it is not yet known whether the well-known Uemura relation holds in pnictide superconductors. Several earlier muon-spin-relaxation (*μ*SR) studies claimed that *T*_*c*_ scales with the superfluid density^35,36^. On the other hand, a recent *μ*SR study refuted such claims^37^.

In a compound with both electron and hole pockets at the Fermi surface, the resistivity can be effectively described as *ρ* = [(*n*_*e*_ *μ*_*e*_ + *n*_*h*_ *μ*_*h*_)*e*]^−1^, where *n*_*e*_(*n*_*h*_) and *μ*_*e*_(*μ*_*h*_) represent the carrier density and the mobility of the electron (hole), respectively. In such case, the nature of *ρ*(*T*) curve gets affected when the mobility of either the electron pocket or hole pocket changes due to some external perturbation. The Fermi surface of the undoped 1111 compound consists of a large hole pocket at the Γ point, mostly from the *d*_*yz*_ and *d*_*zx*_ states of Fe and a smaller electron pocket at the *M* point, mainly contributed by *d*_*xy*_ and *d*_*yz*/*zx*_ orbitals of Fe^38–42^. With increasing electron doping, the size of electron pocket gradually increases and the hole pocket is shifted below the Fermi level. Application of pressure mainly influences the band structure directly by increasing the bandwidth, which implies changes in the carrier effective mass, band overlap and electron correlation. Investigations in La-1111^20^, Sm-1111^43^ and Co-doped Ba-122^44^ have indicated that pressure modifies the shape of the electron pocket at *M* point and increases its influence on transport properties, while the contribution from the hole pocket remains unchanged.

In summary, we have investigated the effect of high pressure up to 8 GPa on both superconducting and normal state properties of optimally doped oxygen-deficient PrFeAsO_0.6_F_0.12_ sample in which sharp superconducting transition and large superconducting volume fraction are observed. With increase in pressure, *T*_*c*_ initially increases for pressure up to 1.3 GPa and then decreases. The Meissner signal shows a systematic increase with pressure up to 1.1 GPa. In the normal-state, pressure suppresses resistivity monotonically up to the highest applied pressure 8 GPa. The nonmonotonic pressure dependence of *T*_*c*_ reveals a dome-shaped (*T*_*c*_-*P*) phase diagram for PrFeAsO_0.6_F_0.12_ with two distinct regions. On the other hand, both *T*_*c*_ and Meissner signal are observed to decrease with pressure for overdoped PrFeAsO_0.6_F_0.14_ sample.

## Methods

Single-phase polycrystalline PrFeAsO_0.6_F _*y*_ samples, where both fluorine doping and oxygen-deficiency are present, have been prepared by conventional solid-state reaction using high-purity chemicals from Alfa-Aesar. Fine powders of Pr_(1-y/3)_As, Fe, Fe_2_O_3_, PrF_3_ and Pr_6_O_11_ were mixed in appropriate ratios, pressed into pellets and then wrapped with tantalum foil and sealed in an evacuated quartz tube. These pellets were heated at 1250–1275 °C for 36 h and then at 1150 °C for about 24 h with an intermediate grinding. The phase purity and chemical homogeneity of the sample were determined by the powder x-ray diffraction and energy dispersive x-ray analysis, respectively^31,45^. We observe that the grains are chemically homogeneous. High-magnification SEM images show plate-like crystallites with a size of 2–30 *μ*m. The electrical resistivity on sample of typical dimensions 0.5 × 0.25 × 0.10 mm^3^ was measured by standard four-probe technique at different pressures in the temperature range 4–300 K. The hydrostatic pressure up to 3 GPa was generated using a self-clamp type hybrid double-cylinder (NiCrAl-inner cylinder; BeCu-outer cylinder) pressure cell. The Daphne 7373 was used as a pressure transmitting medium and the applied pressure was calibrated using a calibration curve that was previously obtained by the observation of fixed-pressure points (Bi) at room temperature^46^. The low-resistance electrical contacts were made by using thin copper wire with high quality silver paint. The high pressure resistivity measurements above 1 GPa were done using a cubic anvil pressure device, consisting of six tungsten carbide anvils, which have been used to produce homogeneous hydrostatic pressure up to 8 GPa^47^. The dc magnetization measurements under pressure were done using physical property measurement system (Quantum Design, USA). The external pressure up to 1.1 GPa was generated by a clamp type miniature hydrostatic pressure cell which is made of nonmagnetic CuBe alloy. The fluorinert FC 70 and FC 77 (1:1) mixture was used as a pressure transmitting medium and the *in-situ* pressure was estimated from the superconducting transition of pure Sn.

## Electronic supplementary material

Supplementary Information
