# Supplementary material for: Effect of pressure on normal and superconducting state properties of iron based superconductor PrFeAsO0.6Fy (y = 0.12, 0.14)
Source: Sci Rep. 2017 Sep 15;7:11731. doi: 10.1038/s41598-017-11927-1 (PMC5601470; doi:10.1038/s41598-017-11927-1)
Supplement: Supplementary file 1 — Supplementary Information [file 41598_2017_11927_MOESM1_ESM.pdf]

Supplementary Information for "Effect of pressure on normal and superconducting state properties of iron based superconductor $PrFeAsO_{0.6}F_y$ (y=0.12, 0.14)"

S. Arumugam[1], C. Ganguli[2], R. Thiyagarajan[1], D. Bhoi[3], G. Kalai Selvan[1], K. Manikandan[1], A. Pariari[3], P. Mandal[3], and Y. Uwatoko[2]

[1] *Centre for High Pressure Research, School of Physics, Bharathidasan University, Tiruchirappalli 620 024, India*

[2] *ISSP, University of Tokyo, 5-1-5 Kashiwanoha, Kashiwa, Chiba 277-8581, Japan*

[3] *Saha Institute of Nuclear Physics, HBNI, 1/AF Bidhannagar, Calcutta 700 064, India.*

Sample Characterization:

The phase purity of both $PrFeAsO_{0.6}F_{0.12}$ and $PrFeAsO_{0.6}F_{0.14}$ samples was determined by high resolution powder x-ray diffraction using a Rigaku x-ray diffractometer (TTRAX II) [30]. The diffraction pattern for $PrFeAsO_{0.6}F_{0.12}$ is shown in Fig. S1 as a representative. We did not observe any impurity phase within the resolution of x-ray for both of our sample $PrFeAsO_{0.6}F_{0.12}$ and $PrFeAsO_{0.6}F_{0.14}$. The evaluated lattice parameters are a = 3.9711 Å and c = 8.5815 Å for $PrFeAsO_{0.6}F_{0.12}$, a = 3.9706 Å and c = 8.5798 Å for $PrFeAsO_{0.6}F_{0.14}$ sample. The observed lattice parameters are close that reported earlier [28,29].

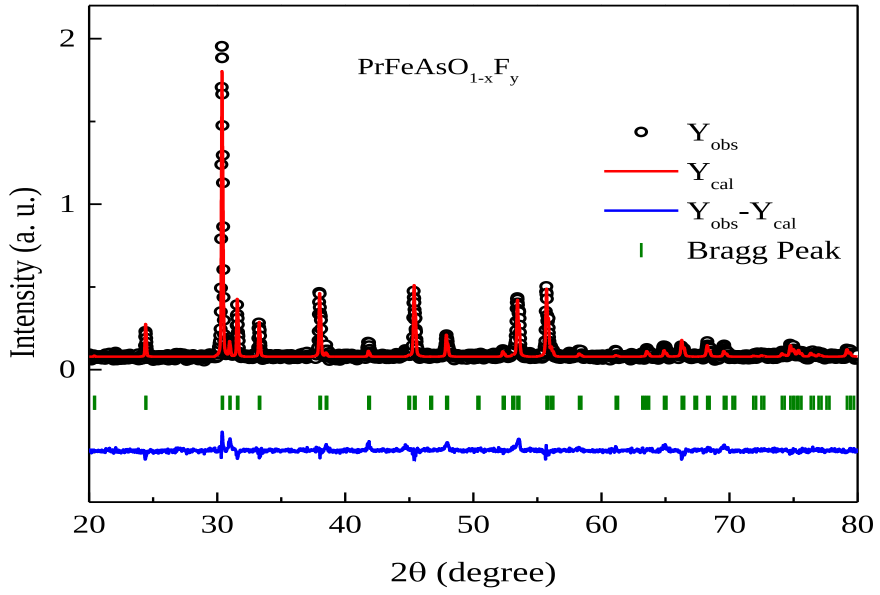

Fig. S1: Reitveld refinement of the powder x-ray data for $PrFeAsO_{0.6}F_{0.12}$ sample at room temperature. The x-ray diffraction peaks can be well indexed on the basis of a tetragonal ZrCuSiAs-type structure with P4/nmm space group.

Ren *et al* prepared oxygen deficient $PrFeAsO_{0.85}$ samples at ambient pressure and also using high pressure technique [28,29]. The lattice parameters for the sample prepared under high pressure are parameter a =3.968 Å and c=8.566 Å while the corresponding values are 3.985 Å and 8.600 Å for samples prepared at ambient condition.

We have done energy dispersive x-ray [EDX] for the determination of chemical composition and homogeneity of the prepared samples [46]. The typical EDX spectrum for the $PrFeAsO_{0.6}F_{0.12}$ sample is shown in Fig. S2: From the EDX spectra, the oxygen content is found to be ~0.75 which is slightly higher than the starting composition. The cations are close to that of nominal composition. Examining the composition at several points, we did not find any local inhomogeneity. This observation suggests that the grains are chemically homogeneous within the limit of SEM analysis.

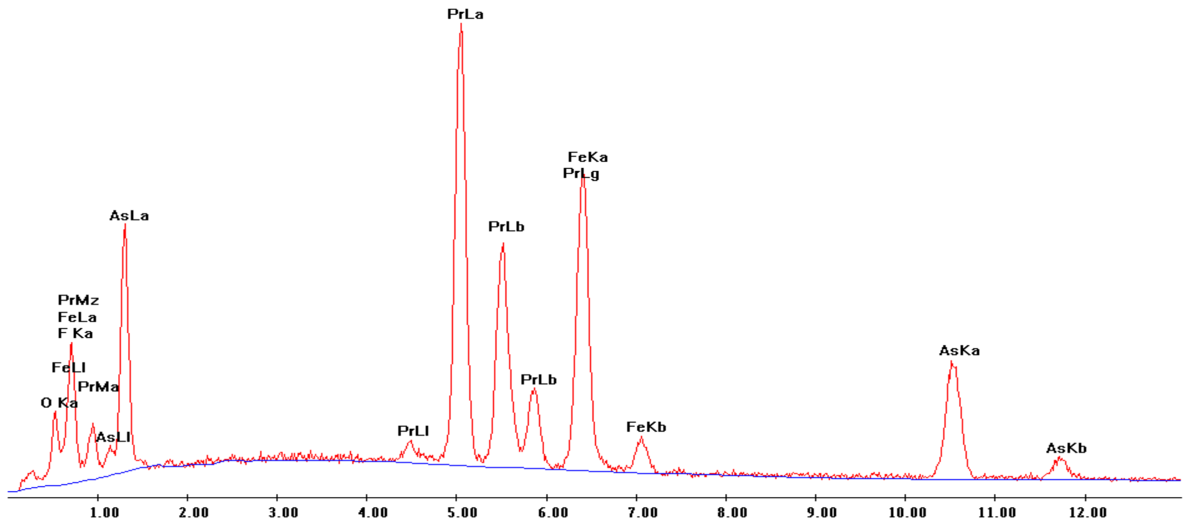

Fig. S2: Energy dispersive X-ray (EDX) spectra of the $PrFeAsO_{0.6}F_{0.12}$ sample.

Hall measurements:

To determine the exact state of doping, the Hall coefficient measurement is much more accurate than the EDX or other chemical methods. Though, EDX is quite accurate in determining the content of heavy ions, it may give significant amount of error for lighter elements. Thus to understand the evolution of carrier concentration with fluorine doping (y) in $PrFeAsO_{0.6}F_y$, it is better to measure to the Hall resistivity in these samples. In Fig. S3, we have plotted the Hall resistivity for both the samples as a function of magnetic field.

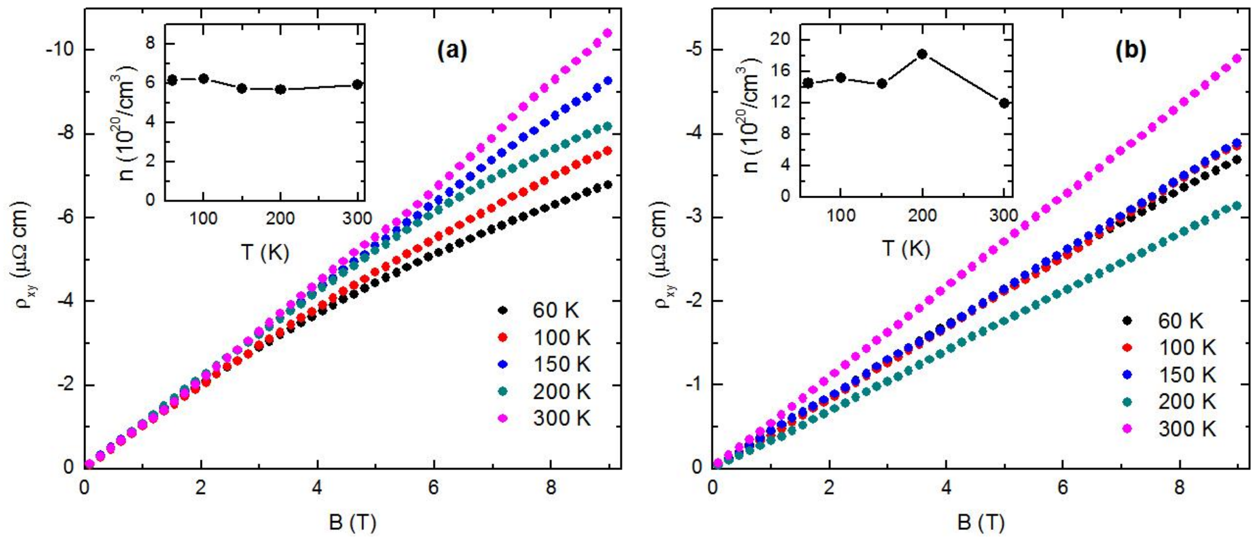

Fig. S3: Hall resistivity (ρ_{xy}) as a function of magnetic field for (a) $PrFeAsO_{0.6}F_{0.12}$ sample and (b) $PrFeAsO_{0.6}F_{0.14}$ sample. Inset shows the corresponding temperature dependence of carrier density.

It is evident from the sign of ρ_{xy}, as shown in Figure S3 that the transport in both the optimal doped and over-doped compounds is dominated by the electron type charge carrier. $\rho_{xy}(B)$ is weakly sublinear for both the optimal doped and over doped compounds. Considering the linear field region and employing the relation, $\rho_{xy} = 1/ne$, the carrier density (n) has been calculated. Insets of Fig. S3(a) and S3(b) show the values n for $PrFeAsO_{0.6}F_{0.12}$ and $PrFeAsO_{0.6}F_{0.14}$ samples, respectively.
